# Hæmoptysis and Nitrite of Amyl

**Published:** 1907-12-28

**Authors:** Francis Hare

**Affiliations:** 93 Church Road, Upper Norwood, S.E.


### Editor's Letter-Box.

### HÆMOPTYSIS AND NITRITE OF AMYL.

Sir,—In his most interesting and comprehensive article on "Some Medical Hæmorrhages" (Hospital, December 14, 1907) Dr. Leonard Williams makes some statements which are certainly open to criticism. He points out that in hæmoptysis the blood may come either from the pulmonary or the bronchial arteries—that is, either from the lesser or the greater circulation. This must, of course, be admitted. But he asserts that the inhalation of nitrite of amyl which causes "widespread dilatation of the systemic vessels, with a considerable and rapid fall of arterial pressure," exerts "no necessary, or even probable, consequential effect on the vessels in the pulmonary system. . . . If the ruptured vessel be in the pulmonary system then nitrite of amyl, though it may do no harm, will certainly do no good. Where the hæmoptysis is due to mitral stenosis it is always a pulmonic vessel which is affected, so that in this condition, at any rate, nitrite of amyl is useless. . . . The utility of the nitrite depends upon which (system of vessels) is affected."

I propose to move the following amendment:—That no matter whether the hæmorrhage comes from the pulmonary or from the systemic vessels, inhalation of nitrite of amyl is equally useful. And this upon both *à priori* and *à posteriori* grounds.

*It is impossible to cause widespread dilatation of the systemic vessels without reducing the pressure in the pulmonary vascular system.* The dilatation of the systemic periphery involves a diminution of resistance, and consequently a fall of blood-pressure. This of necessity travels backwards in the course of the circulation. There is consecutively diminished resistance and fall of blood-pressure (1) in the systemic arteries, (2) in the aortic outflow, (3) in the left ventricle, (4) in the left auricle, and (5) in the pulmonary periphery. This is pointed out by Schäfer (Text-book of Physiology, 1900, vol. ii., p. 150).

*Conversely, any widespread constriction of the systemic vessels will increase the pressure in the pulmonary vascular system.*

Clinical experience is amply confirmatory of both propositions. In an article in the *British Journal of Tuberculosis* for January 1907 I referred to sixty attacks of hæmoptysis occurring in thirty-three patients. All the attacks were treated by inhalation of nitrite of amyl, and in every instance except one the bleeding ceased or became reduced to a mere staining of the sputa, immediately, that is, within a minute or so. In the great majority of these cases the patients were suffering from pulmonary tuberculosis; and it is certain that in some of these, at any rate, the bleeding came from the pulmonary system. Moreover, in the first case in which nitrite of amyl was successfully used, the hæmorrhage almost certainly came from the pulmonary vessels, since the lesion responsible was mitral insufficiency, not tuberculosis (The Food Factor in Disease, 1905, vol ii., p. 94).

There are many clinical facts illustrating the tendency to rise of pressure in the pulmonary vascular system which accompanies any widespread vaso-constriction of the systemic periphery. At the commencement of each menstrual period there is a distinct rise of blood-pressure which is certainly in part, at least, dependent on increased vaso-constriction of the systemic periphery; and it is well known that in tuberculous females there is a special risk of hæmoptysis at each menstrual period. Exposure of the skin to cold—air or water—is apt to determine hæmoptysis in both pulmonary tuberculosis and mitral disease. Adrenalin and ergot have both been shown to cause widespread systemic vaso-constriction with consequent rise of blood-pressure. Associated therewith is engorgement of the pulmonary vascular system (W. E. Dixon, *Lancet*, March 24, 1906, pp. 826, 827). And cases have been reported in which the administration of the former drug seemed to reinduce a hæmoptysis which had ceased (J. Gray Duncanson, *Lancet*, March 12, 1904, p. 603).

Everything goes to show that nitrite of amyl causes anæmia of the pulmonary tissue, not congestion, as was imagined. And if further evidence is demanded, it may be found in direct laboratory experiment. Soulier, Pic, and Petitjean proceed as follows:—A dog is curarised, the anterior portion of the chest wall removed, and life maintained by artificial respiration. The lung thus exposed exhibits the normal rose colour. One cubic centimetre of nitrite of amyl is then injected into the femoral vein. Quickly white patches commence to form on the lung. These increase in size, coalesce, and soon cover the whole surface: the lung has become exsanguine. The process commences ten seconds after the femoral injection. The exsanguine condition endures with an intensity which is often extreme for several minutes before it begins to lessen; nor does the lung resume its normal rose colour for from seven to ten minutes. No reactionary hyperæmia ensues. The intensity of the pulmonary anæmia is gauged by snipping the lung tissue with scissors. In ordinary circumstances profuse hæmorrhage follows such an injury; but in the case of the dog under the influence of nitrite of amyl no single drop of blood issues from the wound (Comptes rendus hebdomadaires des Séances de la Société de Biologie, January 20, 1906, p. 131; also Lyon Médical, February 18, 1906, p. 309 *et seq.*; and thesis by Georges le Bourland, entitled "Traitement des Hémoptysies par le nitrite d'amyl," Lyons, 1905). Now, it is at once manifest that such a degree of anæmia of the lung parenchyma could not be brought about by an action on the bronchial arteries alone.—I am, Sir, Yours faithfully,

Francis Hare.

93 Church Road, Upper Norwood, S.E.

### METROPOLITAN HOSPITAL SUNDAY FUND.

With reference to the report of the annual meeting of the constituents of this fund on the 17th instant, which appeared on page 327 of our issue last week, the name of the Royal Commission on Vivisection was wrongly given in the first edition of our issue as the Anti-Vivisection Committee. The error was corrected in the second edition of last week's issue.

THE HOSPITAL *December* 28, 1907.

